# pEGASUS HPC stent pusher-assisted catheterization (PAC) of nonruptured cerebral aneurysms: Safety and efficacy

**DOI:** 10.1177/15910199251348005

**Published:** 2025-06-10

**Authors:** Bayan Alhaj Moustafa, Ali Khanafer, Mete Dadak, Christopher Nimsky, Alexander Grote, Abdallah Aburub, Hans Henkes, André Kemmling, Mohammad Almohammad

**Affiliations:** 1 Department of Diagnostic and Interventional Neuroradiology, University Hospital Marburg, Marburg, Germany; 2 Department of Neuroradiology, 14881 Klinikum Stuttgart—Katharinenhospital, Stuttgart, Germany; 3 Department of Radiology, St Vincenz Hospital Paderborn, Paderborn, Germany; 4 Department of Neurosurgery, University Hospital Marburg, Marburg, Germany; 5 Department of Radiology, 39515 Rhön-Klinikum, Campus Bad Neustadt, Bad Neustadt an der Saale, Germany

**Keywords:** Stent, pusher, pEGASUS HPC, coil, embolization, perforation

## Abstract

**Objectives:** To investigate the safety and efficacy of using the pEGASUS HPC stent pusher instead of a microwire for catheterization of nonruptured cerebral aneurysms during stent-assisted coiling. **Methods:** In this multicenter retrospective study (July 2021–June 2024), 107 patients with 118 incidental nonruptured cerebral aneurysms underwent stent-assisted coiling using pEGASUS HPC stents. Based on the catheterization technique, cases were assigned to either the microwire-assisted catheterization (MAC, n = 58) or the stent pusher-assisted catheterization (PAC, n = 60) group. Clinical and procedural data were analyzed to compare safety and efficacy, focusing on success rates, required catheterization time, complications, and adverse events. **Results:** The cohort (mean age 59 ± 13.2 years; 52.3% female). In the MAC group, aneurysm catheterization was successful in all cases (100%), with one procedure-related perforation caused by the microwire (1.7%). In contrast, the PAC group achieved a slightly lower success rate of 95% (57/60), but no perforations were observed. The mean catheterization time was significantly shorter in the PAC group (0.67 ± 0.24 minutes) compared to the MAC group (4.43 ± 0.59 minutes), demonstrating that PAC is approximately seven times faster. No other relevant complications were reported. **Conclusion:** PAC with the pEGASUS HPC stent pusher appears safe and effective for catheterizing nonruptured cerebral aneurysms, with high success, no perforations, and significantly shorter catheterization time. Larger prospective studies are needed to confirm these results.

## Introduction

In stent-assisted coiling (SAC), there are two common methods for placing the tip of the coiling microcatheter into the aneurysm sac, depending on the coiling technique. The first method, known as the jailing technique, involves advancing the coiling microcatheter into the aneurysm sac first, followed by deploying a stent in the aneurysm-carrying vessel using a second microcatheter to trap (jail) the coiling catheter between the stent and the vessel wall. The second method consists of first deploying the stent, and then navigating the coiling microcatheter through the struts of the stent into the aneurysm sac.^1,2^ In this study, the second method was used in all cases.

Three distinct mechanisms of iatrogenic aneurysm perforation during endovascular treatment have been identified: perforation caused by the guidewire, the microcatheter, or the embolization coil.^3,4^ The incidence of such complications is reported to range between 2% and 4.4%, with a markedly increased risk observed in small aneurysms—particularly those with a maximum diameter of less than 4 mm.^3,5,6^

Iatrogenic perforation of cerebral aneurysms during endovascular treatment is a critical complication, accompanied by high morbidity and mortality rates of up to 39%.^7^

The pEGASUS HPC stent is a low-profile, self-expanding, open-cell, laser-cut stent, developed by Phenox GmbH in Bochum, Germany, for optimal adaptation to different vessel configurations to treat wide-neck aneurysms, arterial dissections and intracranial stenosis.^8,9^ It can be delivered through the same 0.0165” inner diameter microcatheters used for coiling, eliminating the need for a microcatheter exchange. Additionally, the stent also has a hydrophilic polymer coating (HPC) to reduce thrombogenicity by inhibiting platelet adhesion on the stent surface.^10,11^

Based on our observations, the tip of the pEGASUS HPC stent pusher sometimes slipped through the stent struts into the aneurysm sac on its own while pulling back the pusher, helping us navigate the microcatheter into the aneurysm sac without the need for a microwire. Recognizing the potential of this phenomenon, we started to use this technique intentionally to catheterize the aneurysms, as it seemed promising, especially in terms of safety and timesaving.

The standard approach for catheterizing the aneurysm sac after stent deployment has traditionally involved advancing a microwire through the stent struts to guide the microcatheter into the sac. To date, no alternative techniques have been systematically described. This study introduces a novel, rapid, and safe method for aneurysm catheterization through the stent struts without the use of a microwire—by using the stent pusher as a rail to guide the microcatheter into the aneurysm sac.

## Methods

### Study design

This retrospective multicenter study was conducted at four neurovascular centers, with consecutive data screened between June 2021 and June 2024. Ethical approval was granted by the local Ethics Committee (IRB: 24-180 RS); informed consent was waived due to the retrospective design and anonymized data collection and analysis.

### Study participants

Patients were included if they had one or more incidental, nonruptured cerebral aneurysms treated by SAC using the pEGASUS HPC stent exclusively. Only cases in which the pEGASUS HPC stent was used (one or more per case) were considered. Patients were excluded if other stents or intrasaccular devices were used, if the jailing technique was applied, or if they presented with an already ruptured aneurysm.

### Endovascular procedures

**Figure 1.**
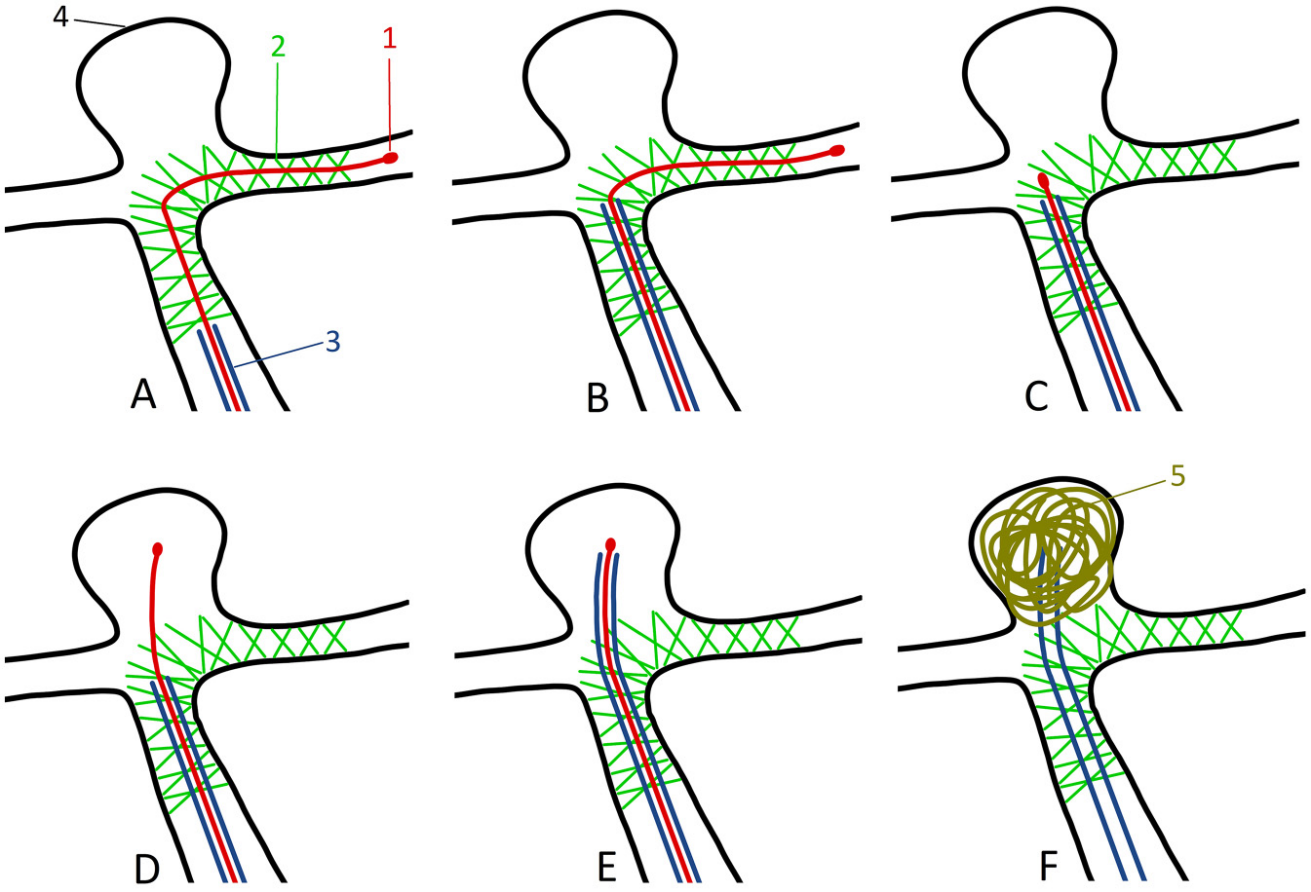
Schematic illustration of the step-by-step pEGASUS stent pusher-assisted catheterization (PAC) technique of a bifurcation aneurysm. (A) Vascular anatomy immediately after stent deployment across the aneurysm neck. 1—Tip of the stent pusher; 2—deployed pEGASUS HPC stent; 3—microcatheter used for stent deployment, which will be navigated into the aneurysm sac and used for coiling; and 4—aneurysm sac. (B) The microcatheter tip is positioned at the aneurysm neck. (C) The stent pusher tip is retracted to the aneurysm neck. (D) The stent pusher tip is advanced through the stent struts into the aneurysm sac. (E) The microcatheter is guided through the stent struts into the aneurysm sac using the stent pusher as a rail. (F) The stent pusher is removed, and coil embolization of the aneurysm is performed.

**Figure 2.**
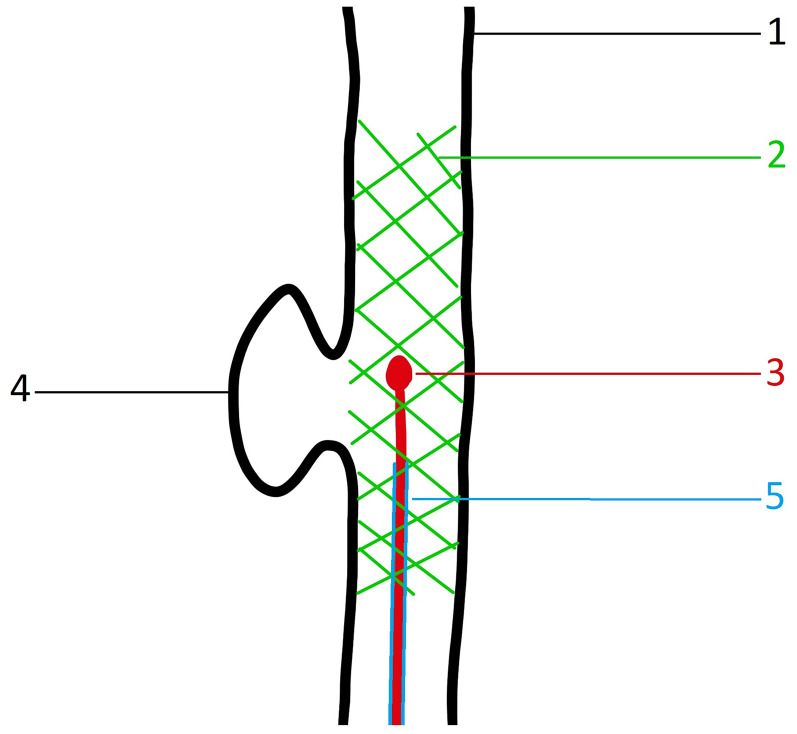
Schematic illustration demonstrating a tangentially located aneurysm relative to its parent vessel, resulting in the failure of the pEGASUS stent pusher-assisted catheterization (PAC) technique. 1—Parent vessel; 2—deployed pEGASUS HPC stent; 3—tip of the stent pusher; 4—aneurysm sac; and 5—microcatheter used for stent deployment, which could not be navigated into the aneurysm sac to perform coiling due to the challenging angulation. In this scenario, a curved microwire was required to access the aneurysm.

**Figure 3.**
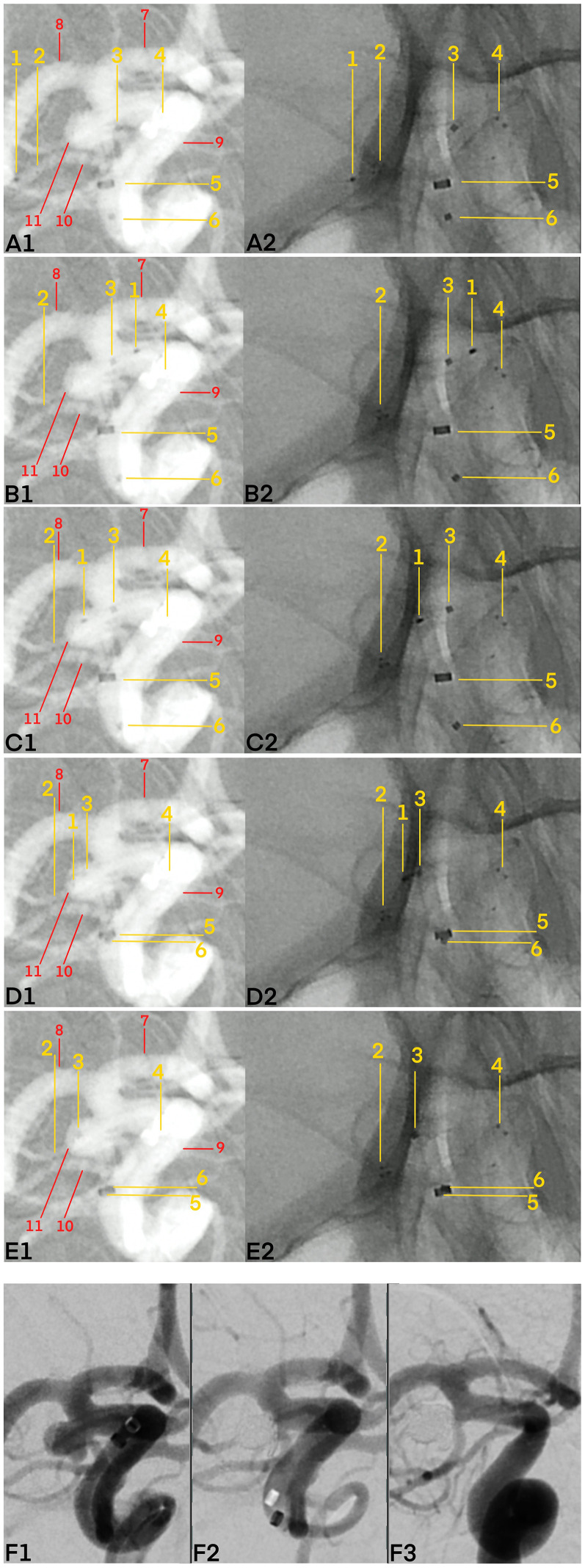
Real case example illustrating the pEGASUS stent pusher-assisted catheterization (PAC) technique in a patient with a saccular aneurysm of the right posterior communicating artery (pComA). Digital subtraction angiography (DSA) demonstrates the step-by-step application of the PAC technique. The following key anatomical landmarks and device components are annotated: 1—Stent pusher tip; 2—distal markers of the deployed pEGASUS stent; 3—microcatheter tip; 4—proximal stent markers; 5—tip of the distal access catheter; 6—proximal microcatheter marker; 7—anterior cerebral artery (ACA); 8—middle cerebral artery (MCA); 9—internal carotid artery (ICA); 10—PComA; and 11—aneurysm sac. (A1) Vascular anatomy following stent deployment from the PComA to the distal ICA; the microcatheter tip is positioned at the aneurysm neck. (A2) Corresponding unsubtracted image. (B1) The stent pusher tip is retracted to the level of the aneurysm neck. (B2) Unsubtracted image. (C1) The stent pusher tip is advanced through the stent struts into the aneurysm sac. (C2) Unsubtracted image. (D1) Using the stent pusher as a rail, the microcatheter is guided through the stent struts into the aneurysm sac. (D2) Unsubtracted image. (E1) After successful catheterization, the stent pusher is withdrawn, leaving the microcatheter tip positioned in the aneurysm sac, ready for coiling. (E2) Unsubtracted image. (F1) Aneurysm before coiling. (F2) Immediate postembolization result after coils insertion. (F3) Follow-up DSA at 3 months demonstrating complete occlusion of the aneurysm sac.

SAC was performed using the pEGASUS HPC stent (phenox GmbH, Bochum, Germany), which was irreversibly and fully deployed in the aneurysm-carrying vessel across the aneurysm neck. The same microcatheter used for stent deployment was subsequently used for coiling, avoiding catheter exchange. Aneurysm catheterization was performed by advancing the microcatheter through the stent struts into the aneurysm sac. Patients were assigned to two groups based on the catheterization technique: in the microwire-assisted catheterization (MAC) group (n = 58), a Synchro^®^ microwire (0.014” × 215 cm, Stryker^®^) was used for navigation; in the pusher-assisted catheterization (PAC) group (n = 60), the pEGASUS HPC stent pusher itself was used to guide the microcatheter into the sac (Figures 1–3).

### Data collection

Anonymized data included patient demographics (age and gender), aneurysm characteristics (size, neck width, location, and number per patient), procedural parameters (catheterization technique, catheterization time, use of microwire or stent pusher, and stent and coil types), and peri-interventional antiplatelet therapy (medication, duration, and response testing). Additional data included intraprocedural complications (aneurysm perforation, vasospasm, and dissection) and technical success of aneurysm catheterization.

### Procedural and safety endpoints

The primary procedural outcomes included aneurysm catheterization success and catheterization time. Catheterization was considered successful if the coiling microcatheter could be navigated through the stent struts and its tip successfully placed within the aneurysm sac. Catheterization time was defined as the interval between stent deployment and successful catheter positioning.

Safety endpoints focused on the incidence of intraprocedural complications. Aneurysm perforation was defined as the unintended passage of the microwire, microcatheter, or coil through the aneurysm wall into the subarachnoid space, accompanied by contrast extravasation visible on angiography. Rapid identification of such perforations is critical to minimize morbidity and mortality.^7,12^ Therefore, as part of the procedural safety protocol, an angiogram using a small amount of contrast agent was routinely performed via the intermediate catheter immediately after aneurysm catheterization. Dissection was defined as an intimal tear resulting in an intramural hematoma and separation of the vessel wall layers, with angiographic features such as a double-barrel lumen, string sign, or intimal flap.^13–15^ Advanced imaging techniques, such as diffusion-weighted imaging (DWI), are currently being investigated for their potential to improve the early detection of arterial dissections.^16^ Temporary vasospasm was defined as a reversible narrowing of the vessel lumen due to contraction of the vascular smooth muscle, confirmed angiographically.^17^ Additional complications not directly related to aneurysm catheterization—such as air embolism, thromboembolism, or distal vessel occlusion—were also assessed; however, none were observed in this cohort.

### Antiplatelet regimen

All patients received dual antiplatelet therapy consisting of prasugrel (10 mg once daily) and acetylsalicylic acid (100 mg once daily), initiated 5 days prior to the endovascular procedure. Platelet inhibition was assessed on the day of the intervention using VerifyNow™. No cases of nonresponse were observed; all patients demonstrated adequate response to the therapy.

### Statistical analysis

Data were cleaned and analyzed using SPSS (IBM, Version 25 for Windows). Normality of continuous variables was assessed using the Shapiro–Wilk test. Categorical variables are presented as frequencies and percentages, and continuous variables as mean ± standard deviation (SD) or median with interquartile range (IQR), depending on data distribution. Group comparisons were performed using the independent Student's t-test for normally distributed continuous variables or the Mann–Whitney U test for non-normally distributed data. Associations between categorical variables were evaluated using the Chi-square test or Fisher's exact test, as appropriate. A two-sided p-value < 0.05 was considered statistically significant.

## Results

### Demographic and clinical characteristics

A total of 108 patients with 118 incidental, unruptured cerebral aneurysms were treated between June 2021 and June 2024 using one or more pEGASUS HPC stents followed by SAC. All procedures were electively treated. The mean patient age was 59 ± 13.2 years (52.3% were female). The mean aneurysm size was 6.6 ± 2.9 mm, with a mean neck width of 4.1 ± 1.6 mm. The most common aneurysm location was the anterior communicating artery (AComA, n = 39), followed by the middle cerebral artery (MCA, n = 32), posterior communicating artery (PComA n = 19), and basilar artery (BA) tip (n = 12). Less frequent locations included the intracranial internal carotid artery (ICA, n = 8), BA branches (n = 6), and A2 segment of the anterior cerebral artery (ACA, n = 2) (Table 1).

**Table 1.** Demographic and clinical characteristics.

	All (n = 108)	MAC group (n = 58)	PAC group (n = 60)
Age, years (mean ± SD)	59 ± 13.2	59.2 ± 12.9	58.8 ± 13.5
Female sex, n (%)	56 (52.3)	31 (53.4)	25 (41.7)
Male sex, n (%)	52 (47.7)	27 (46.6)	35 (58.3)
Number of aneurysms	118	63	55
Aneurysm size, mm (mean ± SD)	6.6 ± 2.9	6.7 ± 3.0	6.5 ± 2.8
Neck width, mm (mean ± SD)	4.1 ± 1.6	4.2 ± 1.5	4.0 ± 1.7
AComA aneurysms, n (%)	39 (33.1)	20 (31.7)	19 (34.5)
MCA aneurysms, n (%)	32 (27.1)	17 (27.0)	15 (27.3)
PComA aneurysms, n (%)	19 (16.1)	11 (17.5)	8 (14.5)
Basilar tip aneurysms, n (%)	12 (10.2)	6 (9.5)	6 (10.9)
Intracranial ICA aneurysms, n (%)	8 (6.8)	4 (6.3)	4 (7.3)
Basilar branch aneurysms, n (%)	6 (5.1)	3 (4.8)	3 (5.5)
A2 ACA aneurysms, n (%)	2 (1.7)	2 (3.2)	0 (0)

ACA, anterior cerebral artery; AComA: anterior communicating artery; ICA: internal carotid artery; MAC: microwire-assisted catheterization; MCA: middle cerebral artery; PAC: pEGASUS HPC stent pusher-assisted catheterization; PComA, posterior communicating artery; SD: standard deviation.

### Procedural complications

In the MAC group, one case of intraprocedural aneurysm perforation was observed (1.7%). The perforation occurred when the microcatheter, with the microwire extended approximately 3 mm beyond its tip, unintentionally advanced through the aneurysm wall into the subarachnoid space due to sudden tension release from the triaxial system. The complication was managed using a previously described bridging coiling technique^18^: coiling was initiated within the subarachnoid space, and a coil was deployed to bridge the perforation while the microcatheter was carefully retracted into the aneurysm sac, allowing for continued embolization. The patient experienced a small subarachnoid hemorrhage without neurological deficits apart from transient headache and was discharged symptom-free 8 days postintervention. No further treatments such as external ventricular drainage or surgical procedures were required.

Temporary vasospasm was observed in six patients (10.3%) in the MAC group (two in the vertebral artery (VA) and four in the ICA) during navigation of the triaxial system. All cases were managed with intra-arterial infusion of 2 mg nimodipine diluted in 1 L of saline via the guiding catheter, resulting in complete resolution during the procedure. No dissections were reported. In the PAC group, no perforations or dissections occurred. Temporary vasospasm was observed in 8 patients (13.3%; 3 in the VA, 5 in the ICA), all of which responded completely to intra-arterial nimodipine administration, as described above (Table 2).

**Table 2.** Catheterization success and time and complications.

	MAC group (n = 58)	PAC group (n = 60)
Catheterization success, n (%)	58 (100)	57 (95)
Catheterization failure, n (%)	0 (0)	3 (5)
Catheterization time, seconds (mean ± SD)	266 ± 35	40 ± 15
Catheterization time, minutes (mean ± SD)	4.43 ± 0.59	0.67 ± 0.24
Perforations, n (%)	1 (1.7)	0 (0)
Vasospasms, n (%)	6 (10.3)	8 (13.3)
Dissections, n (%)	0 (0)	0 (0)

MAC: microwire-assisted catheterization; PAC: pEGASUS HPC stent pusher-assisted catheterization.

### Catheterization success and time

In the MAC group, aneurysm catheterization was successful in all 58 cases (100%), with a mean catheterization time of 266 ± 35 seconds, corresponding to 4.43 ± 0.59 minutes.

In the PAC group, catheterization was successful in 57 of 60 cases (95%). The mean catheterization time was significantly shorter, at 40 ± 15 seconds, equivalent to 0.67 ± 0.24 minutes. The difference in catheterization success between groups did not reach statistical significance (p = 0.244, Fisher's exact test). In contrast, the difference in catheterization time was statistically significant (p < 0.001, unpaired two-tailed t-test). In all three unsuccessful PAC cases, catheterization was successfully completed using a microwire (Table 2).

## Discussion

This multicenter retrospective cohort study evaluated the safety and efficacy of a novel catheterization technique for nonruptured cerebral aneurysms, in which the pEGASUS HPC stent pusher is used to navigate through the stent struts instead of a conventional microwire, guiding the coiling microcatheter into the aneurysm sac. To our knowledge, this is the first systematic clinical investigation of this approach.

All patients in the cohort received elective, nonemergent treatment with the pEGASUS HPC stent, followed by SAC. The complication rates across both groups were remarkably low, with only one aneurysm perforation among 118 aneurysms (0.8%)—caused by microwire use in the MAC group. No perforations occurred in the PAC group, suggesting a favorable safety profile of the pusher-assisted technique. Temporary vasospasms occurred in both groups with comparable frequency and were unrelated to the use of the pEGASUS pusher, as they were observed before the deployment of the stent in all cases. All vasospasms were managed successfully with intra-arterial nimodipine infusion, and no dissections or additional adverse events were reported.

The PAC technique demonstrated a slightly lower catheterization success rate (95% vs. 100% in MAC), although this difference was not statistically significant (p = 0.244). Notably, all PAC failures occurred in cases where the aneurysm was located tangentially to the parent vessel, which may have hindered the passive advancement of the pusher through the stent struts (Figure 2). In these cases, conversion to MAC allowed completion of the procedure.

The most prominent advantage of PAC was the significant reduction in catheterization time: PAC achieved access to the aneurysm sac in an average of 0.67 ± 0.24 minutes, compared to 4.43 ± 0.59 minutes in the MAC group (p < 0.001). This sevenfold reduction in time not only simplifies procedural workflow but may also reduce radiation exposure, fluoroscopy time, and contrast load—factors known to influence both patient and operator safety.

These results suggest that the PAC technique may be particularly useful in anatomically favorable configurations, where the pusher naturally aligns with the aneurysm sac. Additionally, the elimination of microwire use reduces intravasal manipulation and could decrease the risk of wall perforation, especially in small or fragile aneurysms.

### Limitations

The retrospective design introduces potential selection bias, highlighting the need for prospective, randomized studies. Although the sample size (n = 118) exceeds that of many comparable aneurysm series, it remains modest and limits the generalizability of the results. Additionally, the analysis was restricted to a single stent system (pEGASUS HPC), and therefore the findings may not be transferable to other devices. Long-term clinical and angiographic outcomes were not evaluated and should be addressed in future research.

## Conclusion

The pEGASUS HPC stent PAC technique offers a safe, effective, and time-efficient alternative to traditional microwire navigation during SAC of nonruptured cerebral aneurysms. While the technique may be anatomically dependent, it holds promise for streamlining the catheterization process and minimizing intraprocedural risks. Future prospective studies with larger cohorts and varied stent systems are needed to confirm and expand upon these findings.

## Abbreviations

ACA: anterior cerebral artery
AComA: anterior communicating artery
BA: basilar artery
DWI: diffusion-weighted imaging
HPC: hydrophilic polymer coating
ICA: internal carotid artery
i.a.: intra-arterial
IRB: institutional review board
IQR: interquartile range
MAC: microwire-assisted catheterization
MCA: middle cerebral artery
PAC: pEGASUS HPC stent pusher-assisted catheterization
PComA: posterior communicating artery
SAC: stent-assisted coiling
SD: standard deviation
VA: vertebral artery
